# ErbB2 signaling epigenetically suppresses microRNA‐205 transcription via Ras/Raf/MEK/ERK pathway in breast cancer

**DOI:** 10.1002/2211-5463.12256

**Published:** 2017-07-06

**Authors:** Takuya Hasegawa, Ryohei Adachi, Hitoshi Iwakata, Takayoshi Takeno, Koji Sato, Toshiyuki Sakamaki

**Affiliations:** ^1^ Department of Public Health Faculty of Pharmaceutical Sciences Niigata University of Pharmacy and Applied Life Sciences Japan

**Keywords:** breast cancer, DNA methylation, ErbB2, microRNA, miR‐205, Raf‐1

## Abstract

We previously reported that microRNA‐205 (miR‐205) is downregulated by overexpression of the receptor tyrosine kinase ErbB2 and that ectopic transfection of miR‐205 precursor decreases ErbB2 tumorigenicity in soft agar. In this study, we further analyzed the regulatory mechanisms linking ErbB2 overexpression and miR‐205 downregulation. In ErbB2‐overexpressing breast epithelial cells, miR‐205 expression was significantly increased by treatment with MEK inhibitor U0126 or PD98059, Raf‐1 inhibitor ZM‐336372, and ERK inhibitor SCH772984, but PI3K inhibitor LY294002 and p38 MAPK inhibitor SB203580 had no effect. We established breast epithelial cells overexpressing RafCAAX, a constitutively active form of Raf‐1, and showed that overexpression of RafCAAX dramatically reduced miR‐205 expression. In RafCAAX‐overexpressing cells, miR‐205 expression was also significantly increased by SCH772984. Moreover, miR‐205 expression was significantly increased by treatment with DNA methyltransferase (DNMT) inhibitor 5‐aza‐2′‐deoxycytidine and expression of several DNMT family members was increased in both ErbB2‐ and RafCAAX‐overexpressing cells. DNA methylation analysis by bisulfite sequencing revealed that the putative miR‐205 promoters were predominantly hypermethylated in both ErbB2‐ and RafCAAX‐overexpressing cells. Reporter activity of the putative miR‐205 promoters was reduced in both ErbB2‐overexpressing and RafCAAX‐overexpressing cells. Together, these findings indicate that ErbB2 signaling epigenetically suppresses miR‐205 transcription via the Ras/Raf/MEK/ERK pathway.

**Abbreviations**
DNMT: DNA methyltransferase
EGF: epidermal growth factor
ERK: extracellular signal‐regulated kinase
MEK: mitogen‐activated protein kinase kinase
miRNA or miR: microRNA
PI3K: phosphatidylinositol‐4,5‐bisphosphate 3‐kinase
RT‐PCR: reverse transcription polymerase chain reaction

Breast cancer is a major public health concern worldwide. Morbidity and mortality rates of breast cancer in Japan are increasing rapidly, although it is still lower than in most of other advanced countries 1, 2. Breast cancer is a complex disease with substantial molecular heterogeneity, and our incomplete molecular understanding of breast cancer most likely leads to this unsatisfactory situation. Recent advances in gene expression profiling have identified six intrinsic molecular subtypes of breast cancer (luminal A, luminal B, ErbB2‐enriched, basal‐like, claudin‐low, and normal‐like) 3. Pathological biomarkers such as estrogen receptor (ER), progesterone receptor (PgR), ErbB2, Ki‐67, cytokeratins, and epidermal growth factor (EGF) receptor are mainly used in clinical situations to define breast cancer subtypes. ER/PgR‐positive cancer, defined as luminal A or B, is amenable to hormonal therapies such as tamoxifen or aromatase inhibitors and associated with good prognosis. ER/PgR‐negative cancer and ErbB2‐positive cancer, defined as ErbB2‐enriched, are associated with more aggressive disease and poor prognosis in the absence of effective treatment. Although novel ErbB2‐targeted agents have been developed since trastuzumab was first approved, resistance to these ErbB2‐targeted therapies is an important clinical challenge in the management of breast cancer 4. Therefore, there is a continuing need for the development of additional therapies against ErbB2‐enriched cancer.

MicroRNA (miRNA) are small endogenous noncoding RNA that regulate gene expression mainly at the post‐transcriptional level after binding to specific complementary sequences in the 3′‐untranslated region of the target mRNA. Numerous miRNA have been identified to be aberrantly expressed during breast cancer progression, and are reported to serve as potential biomarkers. miR‐205 is one of the promising biomarkers that negatively correlate with invasion, metastasis, and poor prognosis in breast cancer 5, 6, 7, 8, 9. miRNA are categorized into two groups in terms of their genomic location: intergenic and intragenic. Intergenic miRNA are located outside of annotated protein‐coding genes and have their own transcriptional units, whereas intragenic miRNA are located within introns of annotated protein‐coding genes considered as their host genes. Although intragenic miRNA are thought to be transcribed together with the host genes 10, 11, there are some reports concluding that intragenic miRNA can also have their own promoters and be transcribed independently 12, 13, 14. It is also reported that miR‐205 is an intragenic miRNA and located in chromosome 1 and between the last intron and the last exon of its host gene, *MIR205HG (LOC642587)*. Although previous analyses demonstrated that miR‐205 could be transcriptionally regulated by both *MIR205HG* promoter and its own proximal promoter 15, 16, 17, 18, 19, 20, it remains unclear how miR‐205 expression is regulated in breast cancer. Not to mention, there are few reports on signaling pathway responsible for miR‐205's regulation in breast cancer.

We have previously reported that miR‐205 is downregulated by ErbB2 overexpression and that ectopic transfection of miR‐205 precursor decreases the ErbB2 tumorigenic ability to grow in soft agar 21. In this study, we further analyzed the mechanisms explaining the link between ErbB2 signaling and miR‐205 downregulation by investigating the downstream pathways of ErbB2 signaling. Here, we demonstrate that ErbB2 signaling epigenetically suppresses miR‐205 transcription via Ras/Raf/MEK/ERK pathway.

## Materials and methods

### Cells

Nontumorigenic human breast epithelial cell line MCF10A was purchased from ATCC (Manassas, VA, USA) and maintained in Dulbecco's modified Eagle's medium (DMEM)/F12 supplemented with 5% horse serum, 20 ng·mL^−1^ EGF, 10 μg·mL^−1^ insulin, and 500 ng·mL^−1^ hydrocortisone. DMEM/F12 was purchased from Thermo Fisher Scientific (Waltham, MA, USA), and EGF, insulin, and hydrocortisone were purchased from Sigma (St. Louis, MO, USA). Human breast carcinoma MDA‐MB‐453 cells and SKBR3 cells were kindly provided by Robert I. Glazer in Georgetown University. Human embryonic kidney cell line 293T was kindly provided by Uda in Niigata University of Pharmacology and Applied Life Sciences. MDA‐MB‐453, SKBR3, and 293T cells were maintained in DMEM (Thermo Fisher Scientific) supplemented with 10% fetal bovine serum (Thermo Fisher Scientific).

### Retroviral vector construction

The RafCAAX fragment (~2.0 kb) was digested out with *Xho*I/*Bam*HI from pCMV‐RafCAAX vector (Takara Bio, Shiga, Japan) and inserted into pMSCV‐neo retroviral vector (Takara Bio) digested with *Xho*I/*Bgl*II to get the retroviral expression vector for RafCAAX (pMSCV‐RafCAAX‐neo). *Xho*I, *Bam*HI, and *Bgl*II were purchased from Roche (Basel, Switzerland).

### Retroviral infection

Retroviral infection was performed as described 21. MSCV retroviruses were prepared by transient cotransfection with pMSCV‐RafCAAX‐neo and amphotropic helper virus, pSV‐A‐MLV into 293T cells, by using calcium phosphate precipitation. MCF10A cells were cultured with fresh retroviral supernatants in the presence of polybrene for 48 h and then subjected to selection by 800 μg·mL^−1^ G418 (Sigma). The resulting cells were termed MCF10A‐RafCAAX.

### RNA isolation and real‐time RT‐PCR

Total RNA was isolated with RNAiso Plus (Takara Bio) following the manufacturer's instruction. For the quantitation of mRNA, 2 μg of the total RNA were reverse‐transcribed using High Capacity cDNA Reverse Transcription Kit (Thermo Fisher Scientific), and subsequently, the PCR amplifications were performed in reaction volumes of 20 μL containing 10 μL SYBR *Premix Ex Taq* II (Perfect Real Time) (Takara Bio), 0.4 μm forward and reverse primers, and 5 μL template cDNA using MJ‐Mini thermal cycler combined with the MiniOpticon Real‐Time PCR detection system (Bio‐Rad, San Francisco, CA, USA). The following primers were used: MIR205HG (forward: 5′‐TTTCACCATGTTGCCCAGAC‐3′; reverse: 5′‐AAAGAACATGAGGCCGGATG‐3′) and β‐actin (forward: 5′‐ATTGCCGACAGGATGCAGA‐3′; reverse: 5′‐GAGTACTTGCGCTCAGGAGGA‐3′). The thermal cycling conditions were an initial denaturation step at 95 °C for 10 s, followed by 40 cycles at 95 °C for 10 s and 60 °C for 30 s. Dissociation curve analysis was also performed for all the samples following the completion of amplification to rule out the presence of nonspecific amplifications. The expression value for MIR205HG was normalized to β‐actin. For the quantitation of miRNA, 10 ng of the total RNA was reverse‐transcribed using TaqMan MicroRNA Reverse Transcription Kit (Thermo Fisher Scientific) with specific primers for hsa‐miR‐205‐5p and RNU48 [TaqMan MicroRNA assays (Thermo Fisher Scientific), Assay ID: 000509 (hsa‐miR‐205‐5p); 001006 (RNU48)]. Subsequently, the PCR amplifications were performed in reaction volumes of 20 μL containing 10 μL TaqMan 2X Universal PCR Master Mix, No AmpErase UNG (Thermo Fisher Scientific), 1 μL 20X TaqMan MicroRNA Assay mix (Thermo Fisher Scientific), and 1.33 μL template cDNA using MJ‐Mini thermal cycler combined with the MiniOpticon Real‐Time PCR detection system (Bio‐Rad). The thermal cycling conditions were a hot start step at 95 °C for 10 min, followed by 40 cycles at 95 °C for 15 s and 60 °C for 1 min. Relative miRNA expression of miR‐205 was normalized against the endogenous control, RNU48, using the comparative delta‐delta CT method. Bio‐Rad cfx manager Software was used for quantitation analysis.

### Western blotting

Total whole‐cell lysates were separated by SDS/PAGE and transferred electrophoretically to a 0.2‐μm PVDF membrane. After blocked with 5% dry milk in 0.05% PBST (0.05% Tween 20 in PBS), the membranes were probed with the specific primary antibodies. The appropriate HRP‐conjugated secondary antibodies were subsequently used and immunodetection was performed using the SuperSignal West Femto Maximum Sensitivity Substrate (Thermo Fisher Scientific), followed by fluorescence detection by using ChemiDoc XRS‐J image analysis system (Bio‐Rad). Bio‐Rad quantityone software was used for densitometric analysis. The following antibodies were used: 53, a mouse monoclonal antibody to Raf‐1 (BD Biosciences, San Diego, CA, USA); H‐300, a rabbit polyclonal antibody to DNMT1 (Santa Cruz Biotechnology, Dallas, TX, USA); H‐295, a rabbit polyclonal antibody to DNMT3a (Santa Cruz Biotechnology); H‐230, a rabbit polyclonal antibody to DNMT3b (Santa Cruz Biotechnology); c‐ErbB2 (Oncogene Research Products, San Diego, CA, USA); ab4767, a rabbit polyclonal antibody to Raf1 (phospho‐S621) (Abcam, Cambridge, UK); L38C12, a mouse monoclonal antibody to MEK1/2 (Cell Signaling Technology, Danvers, MA, USA); 41G9, a rabbit monoclonal antibody to phospho‐MEK1/2 (Cell Signaling Technology); L34F12, a mouse monoclonal antibody to p44/42 MAPK (Erk1/2) (Cell Signaling Technology); D13.14.4E, a rabbit monoclonal antibody to phospho‐p44/42 MAPK (Erk1/2) (Cell Signaling Technology); MAB1501, a mouse monoclonal antibody to actin (Millipore, Billerica, MA, USA), as a protein loading control; and horseradish peroxidase‐conjugated goat antibodies to mouse and rabbit IgG (Santa Cruz Biotechnology).

### Signaling pathway inhibition and DNA demethylation

For signaling pathway inhibition experiments, cells were treated with vehicle (0.1% DMSO) or PI3K inhibitor LY294002 (50 μm) (Promega, Madison, WI, USA), p38 MAPK inhibitor SB203580 (10 μm) (Promega), MEK inhibitor U0126 (10 μm) (Promega), PD98059 (20 μm) (Promega), Raf‐1 kinase inhibitor ZM‐336372 (1‐5 μm) (Enzo Life Sciences, Farmingdale, NY, USA), or ERK inhibitor SCH772984 (1 μm) (AdooQ BioScience, Irvine, CA, USA) for 48 h. For DNA demethylation experiments, cells were treated with vehicle (0.1% ethanol) or 5 μm 5‐aza‐2′‐deoxycytidine (Sigma) for 48 h.

### Bisulfite sequencing analysis

Genomic DNA was purified from MCF10A‐ErbB2, MCF10A‐RafCAAX, or MCF10A‐neo cells by using DNeasy Blood & Tissue kit (Qiagen, Hilden, Germany). Bisulfite conversion of cytosine to uracil of the genomic DNA was performed with EpiTect Bisulfite kit (Qiagen) following the manufacturer's instruction. After cleanup of the bisulfite‐converted DNA, PCR was performed with the following primers: promoter A (forward: 5′‐TTAAATGTTAGGATAAGTTTTTGGTTG‐3′; reverse: 5′‐AACCTTACACCTAAAACCTTAATCCT‐3′) and promoter B (forward: 5′‐GGAGGTATGGAGTTGATAATTATGAG‐3′; reverse: 5′‐ACTATCTCTATTCCTAAATCAAAATTACTC‐3′). The PCR amplifications were performed in reaction volumes of 20 μL containing 2 μL 10 × EpiTaq PCR buffer Mg^2+^ free), 0.4 μm forward and reverse primers, 0.3 mm dNTP mixture, 2.5 mm MgCl_2_, 0.025 U·μL^−1^ TaKaRa EpiTaq HS (Takara Bio), and 2 μL template genomic DNA using MJ‐Mini thermal cycler. The thermal cycling conditions were an initial denaturation step at 95 °C for 10 min, followed by 45 cycles at 95 °C for 30 s, 55 °C for 30 s and 72 °C for 30 s, and final extension at 72 °C for 10 min. The final PCR products were cloned into the T‐Vector pMD20 (Takara Bio), and about 10 clones per promoter were sequenced by Sigma‐Genosys (The Woodlands, TX, USA). The DNA methylation level was scored as percentage methylation of individual CpG units by using Quma (http://quma.cdb.riken.jp/).

### Reporter plasmid construction and reporter assay

Genomic DNA was purified from MCF10A cells by using DNeasy Blood & Tissue kit (Qiagen). PCR was performed with the following primers: promoter A (forward: 5′‐TTAAATGCCAGGATAAGCCTCTGGCTG‐3′; reverse: 5′‐AGCCTTGCACCTGAAGCCTTAGTCCT‐3′) and promoter B (forward: 5′‐GGAGGCATGGAGCTGACAACCATGAG‐3′, reverse: 5′‐CTGTCTCTATTCCTAAGTCAGAGTTACTC‐3′). The PCR amplifications were performed in reaction volumes of 50 μL containing 10 μL 5 × PrimeSTAR buffer (Mg^2+^ plus), 0.3 μm forward and reverse primers, 0.2 mm dNTP mixture, 4% DMSO, 0.025 U μL^−1^ PrimeSTAR HS DNA polymerase (Takara Bio), and 5 μL template genomic DNA using MJ‐Mini thermal cycler. The thermal cycling conditions were as follows: 30 cycles of denaturation at 98 °C for 10 s, annealing at 55 °C for 5 s, and extension at 72 °C for 20 s. The final PCR products were inserted into pGL4.14 vector (Promega) digested with *Eco*RV (Roche). The constructed reporter plasmids were transfected using Lipofectamine 3000 reagent (Thermo Fisher Scientific) into MCF10A‐ErbB2, MCF10A‐RafCAAX, or MCF10A‐neo cells, together with pGL4.74 *Renilla* luciferase plasmid (Promega). At 36 h post‐transfection, cells were lysed in Passive Lysis Buffer (Promega), and firefly and *Renilla* luciferase reporter activities were determined. The relative firefly luciferase activities were calculated by normalizing transfection efficiencies according to the *Renilla* luciferase activities.

## Results

### Inhibition of Ras/Raf/MEK/ERK signaling increases miR‐205 expression in ErbB2‐overexpressing breast epithelial cells

**Figure 1:**
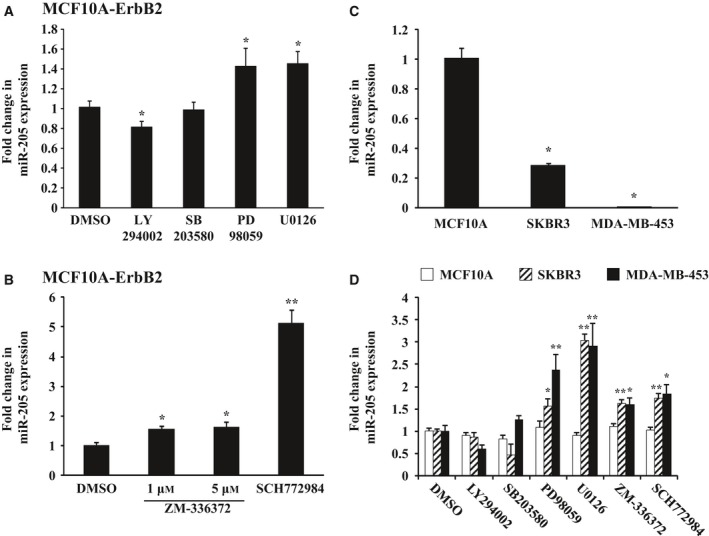
Expression of miR‐205 in MCF10A‐ErbB2, SKBR3, and MDA‐MB‐453 cells treated with ErbB2 signaling pathway inhibitors. Real‐time quantitative RT‐PCR analysis of miR‐205 expression in MCF10A‐ErbB2, SKBR3, and MDA‐MB‐453 cells treated with the indicated inhibitors. (A, D) Cells were treated with PI3K inhibitor LY294002 (50 μm), p38 MAPK inhibitor SB203580 (10 μm), MEK inhibitor U0126 (10 μm), or PD98059 (20 μm) for 48 h. (B, D) Cells were treated with Raf‐1 kinase inhibitor ZM‐336372 (1–5 μm) or ERK inhibitor SCH772984 (1 μm) for 48 h. Data are normalized to DMSO control and represented as mean ± SEM of three independent experiments. *P < 0.05, **P < 0.01 by Student's *t*‐test compared with DMSO. (C) Analysis in MCF10A, SKBR3, and MDA‐MB‐453 cells Data are mean ± SEM of three independent experiments. *P < 0.01 by Student's *t*‐test compared with MCF10A.

We previously reported that downregulation of miR‐205 may be the key event for ErbB2‐induced breast tumorigenesis, but it remains to be determined how ErbB2 signaling can downregulate miR‐205. Thus, we examined the effects of ErbB2 signaling pathway inhibitors on miR‐205 expression in ErbB2‐overexpressing breast epithelial cells in order to determine which ErbB2 downstream pathway is critical for the miR‐205 downregulation. ErbB2‐overexpressing breast epithelial cells were cultured in DMEM/F12 supplemented with 5% horse serum, 20 ng·mL^−1^ EGF, 10 μg·mL^−1^ insulin, and 500 ng·mL^−1^ hydrocortisone for 24 h and then subjected to inhibitor treatment by PI3K inhibitor LY294002, p38 MAPK inhibitor SB203580, MEK inhibitor U0126, PD98059, or vehicle (DMSO). After 48 h, total RNA was purified from the cells and analyzed for miR‐205 expression by real‐time quantitative RT‐PCR. As a result, MEK inhibitors U0126 and PD98059 significantly increased miR‐205 expression in ErbB2‐overexpressing breast epithelial cells compared with vehicle condition, whereas PI3K inhibitor LY294002 and p38 MAPK inhibitor SB203580 had no increasing effects (Fig. 1A). In order to further confirm this observation, we next treated cells with ZM‐336372 (1–5 μm), which is a specific inhibitor of Raf‐1, the upstream activator of MEK, or SCH772984 (1 μm), which is a specific inhibitor of ERK, the downstream effector of MEK. As we had expected, both ZM‐336372 and SCH772984 significantly increased the expression of miR‐205 in ErbB2‐overexpressing breast epithelial cells (Fig. 1B). We also obtained the similar results using other ErbB2‐overexpressing breast cancer cell lines (Fig. S1), MDA‐MB‐453 and SKBR3 in both of which miR‐205 expression was reduced compared with MCF10A (Fig. 1C,D). We also confirmed the effects of those inhibitors on Raf/MEK/ERK phosphorylation by western blotting (Figs S2–S4). Together, these data indicate that ErbB2 signaling decreases miR‐205 expression through Ras/Raf/MEK/ERK signaling pathway.

### Overexpression of RafCAAX decreases miR‐205 expression in breast epithelial cells, and inhibition of ERK increases miR‐205 expression in RafCAAX‐overexpressing cells

**Figure 2:**
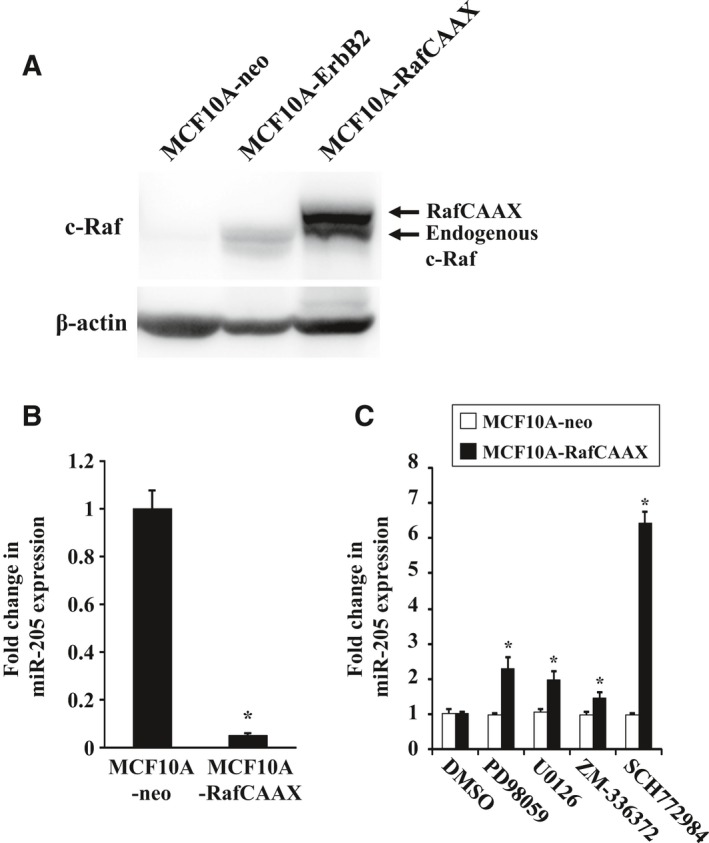
Expression of miR‐205 in MCF10A‐RafCAAX cells treated with ERK inhibitor. (A) Western blot analysis of MCF10A‐RafCAAX, MCF10A‐ErbB2, and MCF10A‐neo cells with anti‐Raf‐1 antibodies. β‐Actin was used as a control for loading. (B) Real‐time quantitative RT‐PCR analysis of miR‐205 expression in MCF10A‐RafCAAX and MCF10A‐neo cells. Data are mean ± SEM of three independent experiments. (C) Real‐time quantitative RT‐PCR analysis of miR‐205 expression in MCF10A‐neo and MCF10A‐RafCAAX cells treated with MEK inhibitor U0126 (10 μm) or PD98059 (20 μm), Raf1 kinase inhibitor ZM‐336372 (1 μm), or ERK inhibitor SCH772984 (1 μm) for 48 h. Data are mean ± SEM of three independent experiments. *P < 0.01 by Student's *t*‐test compared with MCF10A‐neo.

We next established breast epithelial cells stably overexpressing RafCAAX, a constitutively active form of Raf‐1. We constructed the retroviral vector expressing RafCAAX (pMSCV‐RafCAAX‐neo) as described in Materials and methods, and nontumorigenic human breast epithelial cells, MCF10A, were infected by the retroviruses and subsequently subjected to antibiotics selection using G418. The sequences of pMSCV‐RafCAAX‐neo were confirmed by DNA sequencing analysis performed by Sigma‐Genosys. Western blotting analysis showed that the G418‐selected MCF10A cells exhibited overexpression of the active form of Raf‐1 as well as ErbB2‐overexpressing cells (Fig. 2A). We next examined whether overexpressing RafCAAX could affect the expression of miR‐205. As a result of the quantitative RT‐PCR analysis, miR‐205 was reduced by about 95% in expression in RafCAAX‐overexpressing cells compared with vector control stable cells established before as described 21 (Fig. 2B). Moreover, treatment with PD98059, U0126, ZM‐336372, and SCH772984 significantly increased the expression of miR‐205 in RafCAAX‐overexpressing breast epithelial cells, but not in control MCF10A‐neo cells (Fig. 2C). However, the increasing effects of ZM‐336372 were not so striking in MCF10A‐ErbB2 and MCF10A‐RafCAAX cells compared with those of SCH772984. We previously reported that ErbB2 siRNA transfection in MCF10A‐ErbB2 cells decreased ErbB2 expression by 80% but increased miR‐205 expression by just twofold 21. ZM‐336372 treatment increased miR‐205 expression at least to a similar extent as ErbB2 siRNA transfection. Moreover, SCH772984 treatment inhibited Raf phosphorylation as well as ERK phosphorylation in MCF10A‐ErbB2 and MCF10A‐RafCAAX cells (Figs S4, S5). This may explain that SCH772984 showed larger effects in MCF10A‐ErbB2 and MCF10A‐RafCAAX cells. SCH772984 showed smaller effects in SKBR3 and MDA‐MB‐453 cells in which SCH772984 did not show inhibitory effects of Raf phosphorylation (Fig. S5).

### ErbB2 signaling via Ras/Raf/MEK/ERK pathway decreases miR‐205 expression by DNA methylation

**Figure 3:**
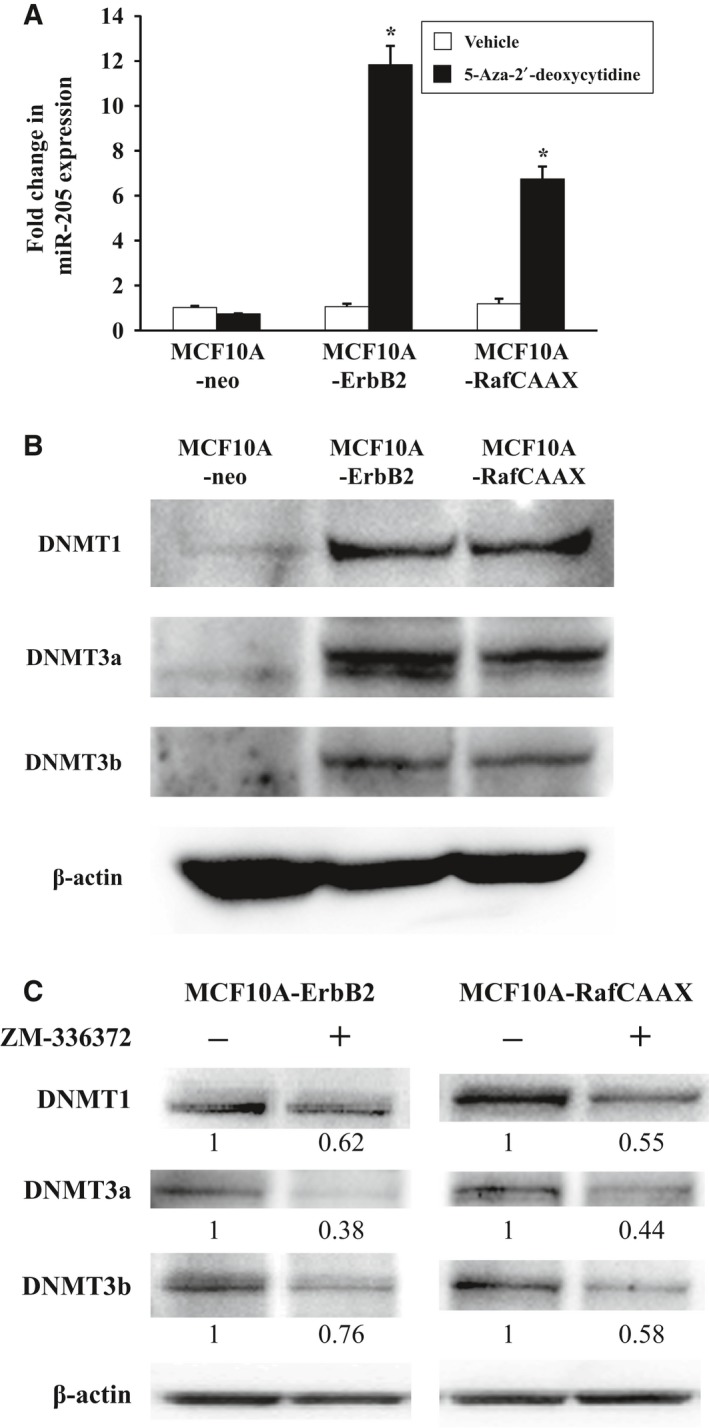
Effects of DNA demethylation on miR‐205 expression and expression of DNMT family in MCF10A‐neo, MCF10A‐ErbB2, and MCF10A‐RafCAAX cells. (A) Real‐time quantitative RT‐PCR analysis of miR‐205 in MCF10A‐RafCAAX, MCF10A‐ErbB2, and MCF10A‐neo cells treated with the DNA methylation inhibitor. Cells were treated with 5‐aza‐2′‐deoxycytidine (5 μm) for 48 h. Data are normalized to vehicle control and represented as mean ± SEM of three independent experiments. *P < 0.01 by Student's *t*‐test compared with vehicle. (B) Western blot analysis of MCF10A‐RafCAAX, MCF10A‐ErbB2, and MCF10A‐neo cells, with antibodies for the indicated proteins. β‐Actin was used as a control for loading. (C) Western blot analysis of ZM‐336372‐treated MCF10A‐ErbB2 and MCF10A‐RafCAAX cells, with antibodies for the indicated proteins. β‐Actin was used as control for loading. Numbers below bands indicate the relative levels of DNMT1, 3a, or 3b normalized to β‐actin, calculated assuming level of untreated cells equal 1, as determined by densitometric analysis.

We next examined how ErbB2 signaling downregulated miR‐205 expression via Ras/Raf/MEK/ERK pathway. Previous studies showed that *ERBB2* amplification frequently associates with DNA hypermethylation in human breast cancers 22. Other studies also have shown a number of hypermethylated genes in ErbB2‐positive breast cancer cells 23, 24, 25. In addition, DNA hypermethylation‐associated repression of tumor suppressor genes has been reported to be essential for Ras‐mediated transformation 26, 27, 28, 29. On the other hand, accumulating evidence shows that miRNA can be deregulated by epigenetic alterations such as aberrant DNA methylation in human cancer, including breast cancer 30, 31, 32, 33, 34, 35, 36, 37, 38, 39. Therefore, to test whether epigenetic alterations affect expression of miR‐205, we treated cells with DNA methyltransferase (DNMT) inhibitor 5‐aza‐2′‐deoxycytidine. As a result, 5‐aza‐2′‐deoxycytidine treatment significantly increased miR‐205 expression by about 11.8‐fold in ErbB2‐overexpressing cells and 6.8‐fold in RafCAAX‐overexpressing cells, but did not alter miR‐205 expression in vector control cells (Fig. 3A). These findings indicate that expression of miR‐205 could be downregulated by promoter hypermethylation.

We next examined whether expression of DNA methylation‐related proteins, DNMT1, DNMT3a, DNMT3b, could be affected by ErbB2 signaling via Ras/Raf/MEK/ERK pathway in breast epithelial cells. As a result, in both ErbB2‐overexpressing and RafCAAX‐overexpressing cells, expression of all DNMT family proteins was increased compared with vector control cells (Fig. 3B). Moreover, ZM‐336372 treatment reduced expression of all DNMT family proteins in both ErbB2‐overexpressing and RafCAAX‐overexpressing cells (Fig. 3C).

### ErbB2 signaling via Ras/Raf/MEK/ERK pathway induces DNA hypermethylation of promoters of both miR‐205 itself and its host gene

**Figure 4:**
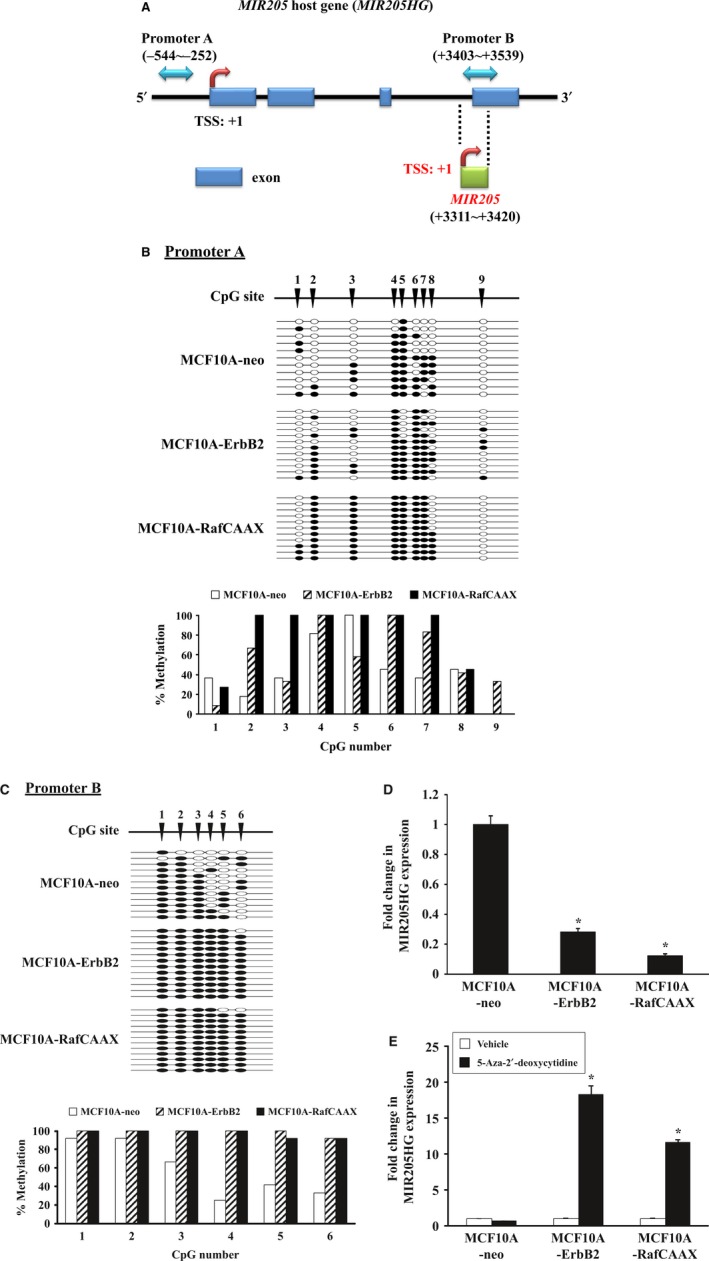
ErbB2 signaling via Ras/Raf/MEK/ERK pathway induces hypermethylation of miR‐205 promoters. (A) Graphical depiction of the *MIR205* host gene (*MIR205HG*). Blue two‐way arrows indicate the promoter regions analyzed by bisulfite sequencing (A: *MIR205HG*, B: *MIR205*). Red bent arrows indicate the putative transcription start sites (TSS) of *MIR205HG* and *MIR205* genes. Blue rectangles represent exons of the *MIR205HG* gene. Green rectangle represents the *MIR205* locus. (B, C) Bisulfite sequencing analysis of promoter A (B) and promoter B (C). White circles indicate nonmethylated cytosine and black circles indicate methylated cytosine at individual CpG sites. The graphs show the percentage of methylation at individual CpG sites. (D) Real‐time quantitative RT‐PCR analysis of MCF10A‐RafCAAX, MCF10A‐ErbB2, and MCF10A‐neo cells for MIR205HG. Data are mean ± SEM of three independent experiments. *P < 0.01 by Student's *t*‐test. (E) Real‐time quantitative RT‐PCR analysis of MIR205HG in MCF10A‐RafCAAX, MCF10A‐ErbB2, and MCF10A‐neo cells treated with DNA methylation inhibitor. Cells were treated with 5‐aza‐2′‐deoxycytidine (5 μm) for 48 h. Data are normalized to vehicle control and represented as mean ± SEM of six independent experiments. *P < 0.01 by Student's *t*‐test compared with vehicle.

In order to further confirm this observation, we analyzed DNA methylation levels of the CpG‐rich regions in both the promoter of MIR205HG and the proximal promoter of miR‐205 by using bisulfite sequencing (Fig. 4A). We chose the promoter regions in our analysis as suggested by previous studies 18, 20. As a result, the bisulfite sequencing revealed increased methylation level in promoter A at CpG sites 2, 4, 6–7 and in promoter B at CpG sites 3–6 in ErbB2‐overexpressing and RafCAAX‐overexpressing cells compared with vector control cells (Fig. 4B,C). In order to further corroborate these results, we also examined the expression of MIR205HG in the same conditions in which miR‐205 repression was seen. As a result, MIR205HG expression was significantly reduced in both ErbB2‐overexpressing and RafCAAX‐overexpressing cells compared with vector control cells (Fig. 4D). In addition, 5‐aza‐2′‐deoxycytidine treatment significantly increased MIR205HG expression in both ErbB2‐overexpressing and RafCAAX‐overexpressing cells (Fig. 4E). Thus, our data indicate that ErbB2 signaling via Ras/Raf/MEK/ERK pathway suppresses miR‐205 transcription through inducing DNA hypermethylation of promoters of both miR‐205 itself and its host gene in breast epithelial cells.

**Figure 5:**
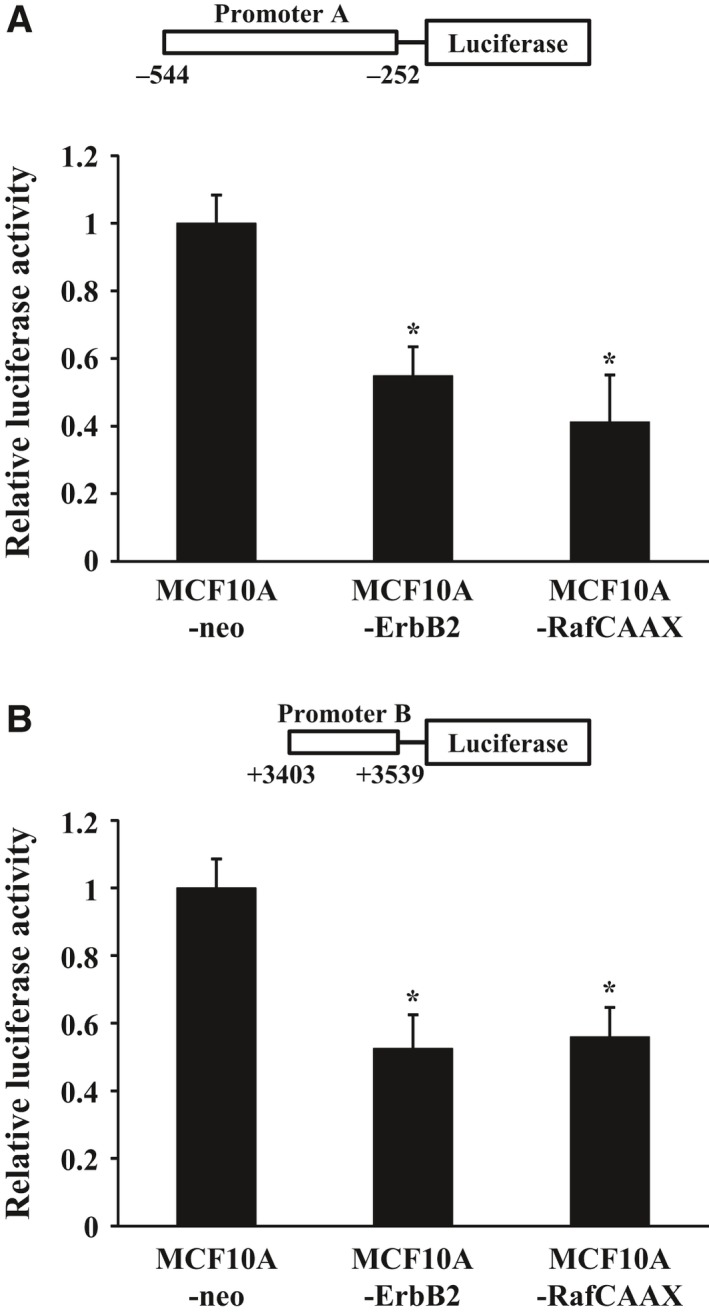
Luciferase reporter analysis of the *miR‐205* promoters. MCF10A‐RafCAAX, MCF10A‐ErbB2, and MCF10A‐neo cells were transiently cotransfected with pGL4.74 *Renilla* luciferase plasmid and reporter plasmid of promoter A (A) or with pGL4.74 *Renilla* luciferase plasmid and reporter plasmid of promoter B (B). At 36 h post‐transfection, luciferase activities were measured. Data are normalized to pGL4.74 *Renilla* luciferase plasmid control and represented as mean ± SEM of three independent experiments. *P < 0.01 by Student's *t*‐test compared with MCF10A‐neo.

We further performed luciferase reporter assays to confirm that ErbB2 signaling via Ras/Raf/MEK/ERK pathway suppresses the activity of miR‐205 promoters. We transfected cells with the reporter plasmids containing either the MIR205HG promoter or the miR‐205 proximal promoter, and analyzed the reporter activities using a dual luciferase detection system. The luciferase activities of the two reporter plasmids were decreased about 50% in both ErbB2‐overexpressing and RafCAAX‐overexpressing cells compared with vector control cells (Fig. 5A,B). Therefore, these data suggest that ErbB2 signaling via Ras/Raf/MEK/ERK pathway regulates the miR‐205 promoters’ activity by altering the DNA methylation level in miR‐205 promoters.

## Discussion

Downstream effectors of proto‐oncogenes can be potential therapeutic targets for cancer treatment. In terms of ErbB2 downstream effectors, mTOR inhibitor, everolimus, is approved in many countries for advanced hormone receptor‐positive, ErbB2‐negative breast cancer in combination with exemestane. In BOLERO‐3, addition of everolimus to trastuzumab plus vinorelbine significantly prolongs progression‐free survival compared with placebo in patients with trastuzumab‐resistant and taxane‐pretreated, ErbB2‐positive, advanced breast cancer 40. We previously reported that miR‐205 might be one of the critical downstream effectors of ErbB2‐induced breast tumorigenesis. Here, we further investigated this hypothesis by analyzing the downstream signaling pathways of ErbB2. In this study, we showed that the inhibitors of Raf‐1, MEK, and ERK significantly increased miR‐205 expression in ErbB2‐overexpressing breast epithelial cells compared with vehicle condition. Moreover, RafCAAX‐overexpressing breast epithelial cells we established in this study showed 95% decrease in miR‐205 expression compared with vector control cells. We also showed that ERK inhibitor significantly increased miR‐205 expression in RafCAAX‐overexpressing cells. Our results suggest that Ras/Raf/MEK/ERK pathway plays a key role in miR‐205 downregulation.

Ras/Raf/MEK/ERK or PI3K/Akt/mTOR pathway is reported to be the main downstream signaling pathway inhibited by trastuzumab 41, 42. Activation of ErbB2 downstream signaling pathways PI3K/AKT/mTOR and Ras/Raf/MEK/ERK contributes to trastuzumab resistance in ErbB2‐positive cancer cells. *PIK3CA* mutation or *PTEN* mutation is implicated in trastuzumab resistance via PI3K/Akt/mTOR pathway activation 43, 44. Elevation of IGF‐1R signaling also contributes to trastuzumab resistance, activating the Ras/Raf/MEK/ERK and PI3K/Akt/mTOR pathways 41, 45. Merry *et al*. recently reported that S100P activates the Ras/Raf/MEK/ERK pathway to compensate for ErbB2 inhibition by trastuzumab 46. Based on these previous findings and our findings, miR‐205 reduction by ErbB2‐downstream Ras/Raf/MEK/ERK signaling might be responsible for trastuzumab resistance. Moreover, miR‐205 upregulation by Ras/Raf/MEK/ERK pathway inhibitors was also seen in MDA‐MB‐453, the cell line with *ErbB2* amplification 47 and concurrent *PTEN* and *PIK3CA* mutations 48, 49. As MDA‐MB‐453 cells are intrinsically resistant to trastuzumab 50, 51 and resistant to everolimus 49, miR‐205 might be a valuable therapeutic target for trastuzumab‐resistant and everolimus‐resistant breast cancer.

**Figure 6:**
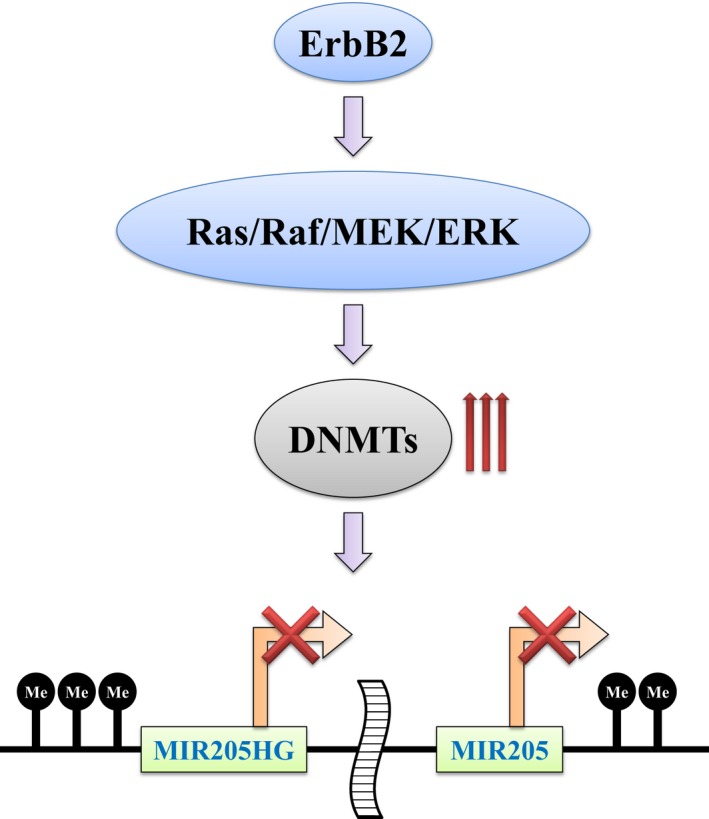
Schematic representation showing downregulation of miR‐205 by ErbB2 signaling via Ras/Raf/MEK/ERK pathway. ErbB2 signaling via Ras/Raf/MEK/ERK pathway leads to hypermethylation of the CpG‐rich regions of the promoters of *MIR205HG* and *MIR205* by inducing DNMT family, resulting in downregulation of miR‐205 expression.

Previous reports indicate that miR‐205 expression is possibly epigenetically regulated in cancer cells such as hepatoma, prostate, bladder, and breast cancer 15, 17, 18, 19, 20. Therefore, we also examined whether miR‐205 expression can be epigenetically modulated by ErbB2 signaling via Ras/Raf/MEK/ERK pathway in breast epithelial cells. Our data obtained in this study allow us to conclude that ErbB2 signaling via Ras/Raf/MEK/ERK pathway suppresses miR‐205 transcription through inducing DNA hypermethylation of the promoters of both miR‐205 itself and its host gene. There are reports that miR‐205 transcription is regulated by both MIR205HG promoter and miR‐205's own proximal promoter 14, 15, 17, 18, 20. Therefore, we examined the methylation status of these two promoters and found that both promoters were hypermethylated in ErbB2‐overexpressing and in RafCAAX‐overexpressing cells. Based on our data, one of the possible mechanisms considered is that ErbB2 signaling via Ras/Raf/MEK/ERK pathway leads to promoter hypermethylation by inducing DNMT family proteins (Fig. 6). However, further analysis needs to be conducted for understanding more details.

We finally want to emphasize that ErbB2 signaling suppresses miR‐205 transcription via Ras/Raf/MEK/ERK pathway. This observation provides novel insights into signaling pathway responsible for miR‐205's regulation in breast cancer.

## Author contributions

TH, KS, and TS conceived and designed the experiments. TH, RA, HI, and TT performed the experiments. TH and TS analyzed the data and wrote the manuscript.

## Supporting information


**Fig. S1.** ErbB2 expression in MCF10A, SKBR3 and MDA‐MB‐453 cells.
**Fig. S2.** Effects of PD98059 or U0126 in MCF10A‐ErbB2, MCF10A‐RafCAAX, MDA‐MB‐453 and SKBR3 cells.
**Fig. S3.** Effects of ZM‐336372 in MCF10A‐ErbB2, MCF10A‐RafCAAX, MDA‐MB‐453 and SKBR3 cells.
**Fig. S4.** Effects of SCH772984 on ERK phosphorylation in MCF10A‐ErbB2, MCF10A‐RafCAAX, MDA‐MB‐453 and SKBR3 cells.
**Fig. S5.** Effects of SCH772984 on c‐Raf phosphorylation in MCF10A‐ErbB2, MCF10A‐RafCAAX, MDA‐MB‐453 and SKBR3 cells.

## References

[1] Desantis CE , Bray F , Ferlay J , Lortet‐Tieulent J , Anderson BO and Jemal A (2015) International variation in female breast cancer incidence and mortality rates. Cancer Epidemiol Biomarkers Prev 24, 1495–1506. 26359465 10.1158/1055-9965.EPI-15-0535

[2] Mizota Y and Yamamoto S (2012) Prevalence of breast cancer risk factors in Japan. Jpn J Clin Oncol 42, 1008–1012. 22988038 10.1093/jjco/hys144

[3] Prat A and Perou CM (2011) Deconstructing the molecular portraits of breast cancer. Mol Oncol 5, 5–23. 21147047 10.1016/j.molonc.2010.11.003 PMC5528267

[4] Gagliato DM , Jardim DL , Marchesi MS and Hortobagyi GN (2016) Mechanisms of resistance and sensitivity to anti‐HER2 therapies in HER2+ breast cancer. Oncotarget 7, 64431–64446. 26824988 10.18632/oncotarget.7043 PMC5325455

[5] Iorio MV , Ferracin M , Liu CG , Veronese A , Spizzo R , Sabbioni S , Magri E , Pedriali M , Fabbri M , Campiglio M *et al* (2005) MicroRNA gene expression deregulation in human breast cancer. Cancer Res 65, 7065–7070. 16103053 10.1158/0008-5472.CAN-05-1783

[6] Luo D , Wilson JM , Harvel N , Liu J , Pei L , Huang S , Hawthorn L and Shi H (2013) A systematic evaluation of miRNA:mRNA interactions involved in the migration and invasion of breast cancer cells. J Transl Med 11, 57. 23497265 10.1186/1479-5876-11-57 PMC3599769

[7] Madden SF , Clarke C , Gaule P , Aherne ST , O'donovan N , Clynes M , Crown J and Gallagher WM (2013) BreastMark: an integrated approach to mining publicly available transcriptomic datasets relating to breast cancer outcome. Breast Cancer Res 15, R52. 23820017 10.1186/bcr3444 PMC3978487

[8] Sempere LF , Christensen M , Silahtaroglu A , Bak M , Heath CV , Schwartz G , Wells W , Kauppinen S and Cole CN (2007) Altered MicroRNA expression confined to specific epithelial cell subpopulations in breast cancer. Cancer Res 67, 11612–11620. 18089790 10.1158/0008-5472.CAN-07-5019

[9] Wu H , Zhu S and Mo YY (2009) Suppression of cell growth and invasion by miR‐205 in breast cancer. Cell Res 19, 439–448. 19238171 10.1038/cr.2009.18 PMC2664859

[10] Baskerville S and Bartel DP (2005) Microarray profiling of microRNAs reveals frequent coexpression with neighboring miRNAs and host genes. RNA 11, 241–247. 15701730 10.1261/rna.7240905 PMC1370713

[11] Rodriguez A , Griffiths‐Jones S , Ashurst JL and Bradley A (2004) Identification of mammalian microRNA host genes and transcription units. Genome Res 14, 1902–1910. 15364901 10.1101/gr.2722704 PMC524413

[12] Corcoran DL , Pandit KV , Gordon B , Bhattacharjee A , Kaminski N and Benos PV (2009) Features of mammalian microRNA promoters emerge from polymerase II chromatin immunoprecipitation data. PLoS One 4, e5279. 19390574 10.1371/journal.pone.0005279 PMC2668758

[13] Ozsolak F , Poling LL , Wang Z , Liu H , Liu XS , Roeder RG , Zhang X , Song JS and Fisher DE (2008) Chromatin structure analyses identify miRNA promoters. Genes Dev 22, 3172–3183. 19056895 10.1101/gad.1706508 PMC2593607

[14] Tran MN , Choi W , Wszolek MF , Navai N , Lee IL , Nitti G , Wen S , Flores ER , Siefker‐Radtke A , Czerniak B *et al* (2013) The p63 protein isoform DeltaNp63alpha inhibits epithelial‐mesenchymal transition in human bladder cancer cells: role of MIR‐205. J Biol Chem 288, 3275–3288. 23239884 10.1074/jbc.M112.408104 PMC3561548

[15] Bhatnagar N , Li X , Padi SK , Zhang Q , Tang MS and Guo B (2010) Downregulation of miR‐205 and miR‐31 confers resistance to chemotherapy‐induced apoptosis in prostate cancer cells. Cell Death Dis 1, e105. 21368878 10.1038/cddis.2010.85 PMC3004480

[16] Gandellini P , Profumo V , Casamichele A , Fenderico N , Borrelli S , Petrovich G , Santilli G , Callari M , Colecchia M , Pozzi S *et al* (2012) miR‐205 regulates basement membrane deposition in human prostate: implications for cancer development. Cell Death Differ 19, 1750–1760. 22555458 10.1038/cdd.2012.56 PMC3469086

[17] Hulf T , Sibbritt T , Wiklund ED , Patterson K , Song JZ , Stirzaker C , Qu W , Nair S , Horvath LG , Armstrong NJ *et al* (2013) Epigenetic‐induced repression of microRNA‐205 is associated with MED1 activation and a poorer prognosis in localized prostate cancer. Oncogene 32, 2891–2899. 22869146 10.1038/onc.2012.300

[18] Lee JY , Park MK , Park JH , Lee HJ , Shin DH , Kang Y , Lee CH and Kong G (2014) Loss of the polycomb protein Mel‐18 enhances the epithelial‐mesenchymal transition by ZEB1 and ZEB2 expression through the downregulation of miR‐205 in breast cancer. Oncogene 33, 1325–1335. 23474752 10.1038/onc.2013.53

[19] Wiklund ED , Bramsen JB , Hulf T , Dyrskjot L , Ramanathan R , Hansen TB , Villadsen SB , Gao S , Ostenfeld MS , Borre M *et al* (2011) Coordinated epigenetic repression of the miR‐200 family and miR‐205 in invasive bladder cancer. Int J Cancer 128, 1327–1334. 20473948 10.1002/ijc.25461

[20] Zhang T , Zhang J , Cui M , Liu F , You X , Du Y , Gao Y , Zhang S , Lu Z , Ye L *et al* (2013) Hepatitis B virus X protein inhibits tumor suppressor miR‐205 through inducing hypermethylation of miR‐205 promoter to enhance carcinogenesis. Neoplasia 15, 1282–1291. 24339740 10.1593/neo.131362 PMC3858896

[21] Adachi R , Horiuchi S , Sakurazawa Y , Hasegawa T , Sato K and Sakamaki T (2011) ErbB2 down‐regulates microRNA‐205 in breast cancer. Biochem Biophys Res Comm 411, 804–808. 21787752 10.1016/j.bbrc.2011.07.033

[22] Terada K , Okochi‐Takada E , Akashi‐Tanaka S , Miyamoto K , Taniyama K , Tsuda H , Asada K , Kaminishi M and Ushijima T (2009) Association between frequent CpG island methylation and HER2 amplification in human breast cancers. Carcinogenesis 30, 466–471. 19168584 10.1093/carcin/bgp021

[23] Fiegl H , Millinger S , Goebel G , Muller‐Holzner E , Marth C , Laird PW and Widschwendter M (2006) Breast cancer DNA methylation profiles in cancer cells and tumor stroma: association with HER‐2/neu status in primary breast cancer. Cancer Res 66, 29–33. 16397211 10.1158/0008-5472.CAN-05-2508

[24] Lindqvist BM , Wingren S , Motlagh PB and Nilsson TK (2014) Whole genome DNA methylation signature of HER2‐positive breast cancer. Epigenetics 9, 1149–1162. 25089541 10.4161/epi.29632 PMC4164500

[25] Yamaguchi T , Mukai H , Yamashita S , Fujii S and Ushijima T (2015) Comprehensive DNA methylation and extensive mutation analyses of HER2‐positive breast cancer. Oncology 88, 377–384. 25591616 10.1159/000369904

[26] Gazin C , Wajapeyee N , Gobeil S , Virbasius CM and Green MR (2007) An elaborate pathway required for Ras‐mediated epigenetic silencing. Nature 449, 1073–1077. 17960246 10.1038/nature06251 PMC2147719

[27] Patra SK (2008) Ras regulation of DNA‐methylation and cancer. Exp Cell Res 314, 1193–1201. 18282569 10.1016/j.yexcr.2008.01.012

[28] Serra RW , Fang M , Park SM , Hutchinson L and Green MR (2014) A KRAS‐directed transcriptional silencing pathway that mediates the CpG island methylator phenotype. eLife 3, e02313. 24623306 10.7554/eLife.02313 PMC3949416

[29] Wu BK and Brenner C (2014) Suppression of TET1‐dependent DNA demethylation is essential for KRAS‐mediated transformation. Cell Rep 9, 1827–1840. 25466250 10.1016/j.celrep.2014.10.063 PMC4268240

[30] Brueckner B , Stresemann C , Kuner R , Mund C , Musch T , Meister M , Sultmann H and Lyko F (2007) The human let‐7a‐3 locus contains an epigenetically regulated microRNA gene with oncogenic function. Cancer Res 67, 1419–1423. 17308078 10.1158/0008-5472.CAN-06-4074

[31] Deneberg S , Kanduri M , Ali D , Bengtzen S , Karimi M , Qu Y , Kimby E , Mansouri L , Rosenquist R , Lennartsson A *et al* (2014) microRNA‐34b/c on chromosome 11q23 is aberrantly methylated in chronic lymphocytic leukemia. Epigenetics 9, 910–917. 24686393 10.4161/epi.28603 PMC4053441

[32] Fazi F , Racanicchi S , Zardo G , Starnes LM , Mancini M , Travaglini L , Diverio D , Ammatuna E , Cimino G , Lo‐Coco F *et al* (2007) Epigenetic silencing of the myelopoiesis regulator microRNA‐223 by the AML1/ETO oncoprotein. Cancer Cell 12, 457–466. 17996649 10.1016/j.ccr.2007.09.020

[33] Jimenez‐Wences H , Martinez‐Carrillo DN , Peralta‐Zaragoza O , Campos‐Viguri GE , Hernandez‐Sotelo D , Jimenez‐Lopez MA , Munoz‐Camacho JG , Garzon‐Barrientos VH , Illades‐Aguiar B and Fernandez‐Tilapa G (2016) Methylation and expression of miRNAs in precancerous lesions and cervical cancer with HPV16 infection. Oncol Rep 35, 2297–2305. 26797462 10.3892/or.2016.4583

[34] Lujambio A , Ropero S , Ballestar E , Fraga MF , Cerrato C , Setien F , Casado S , Suarez‐Gauthier A , Sanchez‐Cespedes M , Git A *et al* (2007) Genetic unmasking of an epigenetically silenced microRNA in human cancer cells. Cancer Res 67, 1424–1429. 17308079 10.1158/0008-5472.CAN-06-4218

[35] Qin J , Ke J , Xu J , Wang F , Zhou Y , Jiang Y and Wang Z (2015) Downregulation of microRNA‐132 by DNA hypermethylation is associated with cell invasion in colorectal cancer. Onco Targets Ther 8, 3639–3648. 26675712 10.2147/OTT.S91560 PMC4676615

[36] Saito Y , Liang G , Egger G , Friedman JM , Chuang JC , Coetzee GA and Jones PA (2006) Specific activation of microRNA‐127 with downregulation of the proto‐oncogene BCL6 by chromatin‐modifying drugs in human cancer cells. Cancer Cell 9, 435–443. 16766263 10.1016/j.ccr.2006.04.020

[37] Scott GK , Mattie MD , Berger CE , Benz SC and Benz CC (2006) Rapid alteration of microRNA levels by histone deacetylase inhibition. Cancer Res 66, 1277–1281. 16452179 10.1158/0008-5472.CAN-05-3632

[38] Yin H , Song P , Su R , Yang G , Dong L , Luo M , Wang B , Gong B , Liu C , Song W *et al* (2016) DNA methylation mediated down‐regulating of microRNA‐33b and its role in gastric cancer. Sci Rep 6, 18824. 26729612 10.1038/srep18824 PMC4700416

[39] Yuan HF , Christina VR , Guo CA , Chu YW , Liu RH and Yan ZQ (2016) Involvement of microRNA‐210 demethylation in steroid‐associated osteonecrosis of the femoral head. Sci Rep 6, 20046. 26805628 10.1038/srep20046 PMC4726266

[40] Andre F , O'regan R , Ozguroglu M , Toi M , Xu B , Jerusalem G , Masuda N , Wilks S , Arena F , Isaacs C *et al* (2014) Everolimus for women with trastuzumab‐resistant, HER2‐positive, advanced breast cancer (BOLERO‐3): a randomised, double‐blind, placebo‐controlled phase 3 trial. Lancet Oncol 15, 580–591. 24742739 10.1016/S1470-2045(14)70138-X

[41] Nahta R , Yu D , Hung MC , Hortobagyi GN and Esteva FJ (2006) Mechanisms of disease: understanding resistance to HER2‐targeted therapy in human breast cancer. Nat Clin Pract Onco 3, 269–280. 10.1038/ncponc0509 16683005

[42] Vu T and Claret FX (2012) Trastuzumab: updated mechanisms of action and resistance in breast cancer. Front Oncol 2, 62. 22720269 10.3389/fonc.2012.00062 PMC3376449

[43] Adamczyk A , Niemiec J , Janecka A , Harazin‐Lechowska A , Ambicka A , Grela‐Wojewoda A , Domagala‐Haduch M , Cedrych I , Majchrzyk K , Kruczak A *et al* (2015) Prognostic value of PIK3CA mutation status, PTEN and androgen receptor expression for metastasis‐free survival in HER2‐positive breast cancer patients treated with trastuzumab in adjuvant setting. Pol J Pathol 66, 133–141. 26247526 10.5114/pjp.2015.53009

[44] Berns K , Horlings HM , Hennessy BT , Madiredjo M , Hijmans EM , Beelen K , Linn SC , Gonzalez‐Angulo AM , Stemke‐Hale K , Hauptmann M *et al* (2007) A functional genetic approach identifies the PI3K pathway as a major determinant of trastuzumab resistance in breast cancer. Cancer Cell 12, 395–402. 17936563 10.1016/j.ccr.2007.08.030

[45] Denduluri SK , Idowu O , Wang Z , Liao Z , Yan Z , Mohammed MK , Ye J , Wei Q , Wang J , Zhao L *et al* (2015) Insulin‐like growth factor (IGF) signaling in tumorigenesis and the development of cancer drug resistance. Genes Dis 2, 13–25. 25984556 10.1016/j.gendis.2014.10.004 PMC4431759

[46] Merry CR , Mcmahon S , Forrest ME , Bartels CF , Saiakhova A , Bartel CA , Scacheri PC , Thompson CL , Jackson MW , Harris LN *et al* (2016) Transcriptome‐wide identification of mRNAs and lincRNAs associated with trastuzumab‐resistance in HER2‐positive breast cancer. Oncotarget 7, 53230–53244. 27449296 10.18632/oncotarget.10637 PMC5288181

[47] Vernimmen D , Gueders M , Pisvin S , Delvenne P and Winkler R (2003) Different mechanisms are implicated in ERBB2 gene overexpression in breast and in other cancers. Br J Cancer 89, 899–906. 12942124 10.1038/sj.bjc.6601200 PMC2394491

[48] Ebi H , Costa C , Faber AC , Nishtala M , Kotani H , Juric D , Della Pelle P , Song Y , Yano S , Mino‐Kenudson M *et al* (2013) PI3K regulates MEK/ERK signaling in breast cancer via the Rac‐GEF, P‐Rex1. Proc Natl Acad Sci U S A 110, 21124–21129. 24327733 10.1073/pnas.1314124110 PMC3876254

[49] Weigelt B , Warne PH and Downward J (2011) PIK3CA mutation, but not PTEN loss of function, determines the sensitivity of breast cancer cells to mTOR inhibitory drugs. Oncogene 30, 3222–3233. 21358673 10.1038/onc.2011.42

[50] Lin XL , Wang XL , Ma B , Jia J , Yan Y , Di LJ , Yuan YH , Wan FL , Lu YL , Liang X *et al* (2012) HER2‐specific T lymphocytes kill both trastuzumab‐resistant and trastuzumab‐sensitive breast cell lines in vitro. Chin J Cancer Res 24, 143–150. 23357961 10.1007/s11670-012-0143-6 PMC3555268

[51] Shim JS , Rao R , Beebe K , Neckers L , Han I , Nahta R and Liu JO (2012) Selective inhibition of HER2‐positive breast cancer cells by the HIV protease inhibitor nelfinavir. J Natl Cancer Inst 104, 1576–1590. 23042933 10.1093/jnci/djs396 PMC3472971

