# Supplementary material for: ErbB2 signaling epigenetically suppresses microRNA‐205 transcription via Ras/Raf/MEK/ERK pathway in breast cancer
Source: FEBS Open Bio. 2017 Jul 6;7(8):1154–65. doi: 10.1002/2211-5463.12256 (PMC5537069; doi:10.1002/2211-5463.12256)

Supplementary Figure S1

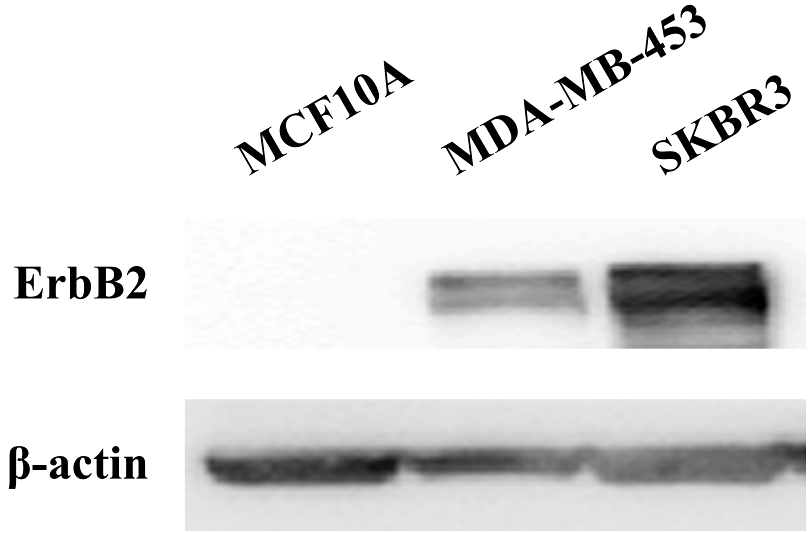

Supplementary Figure S2

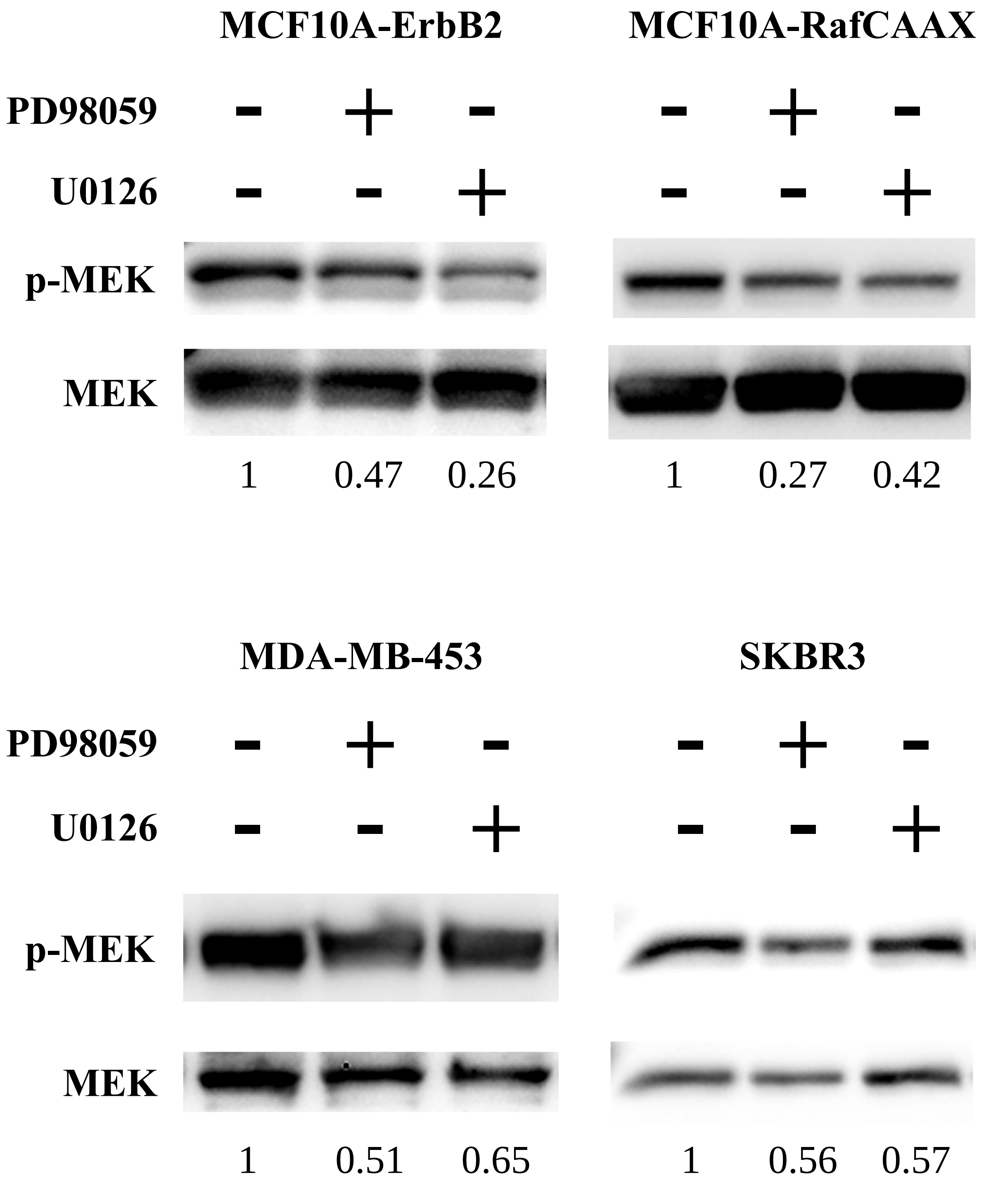

Supplementary Figure S3

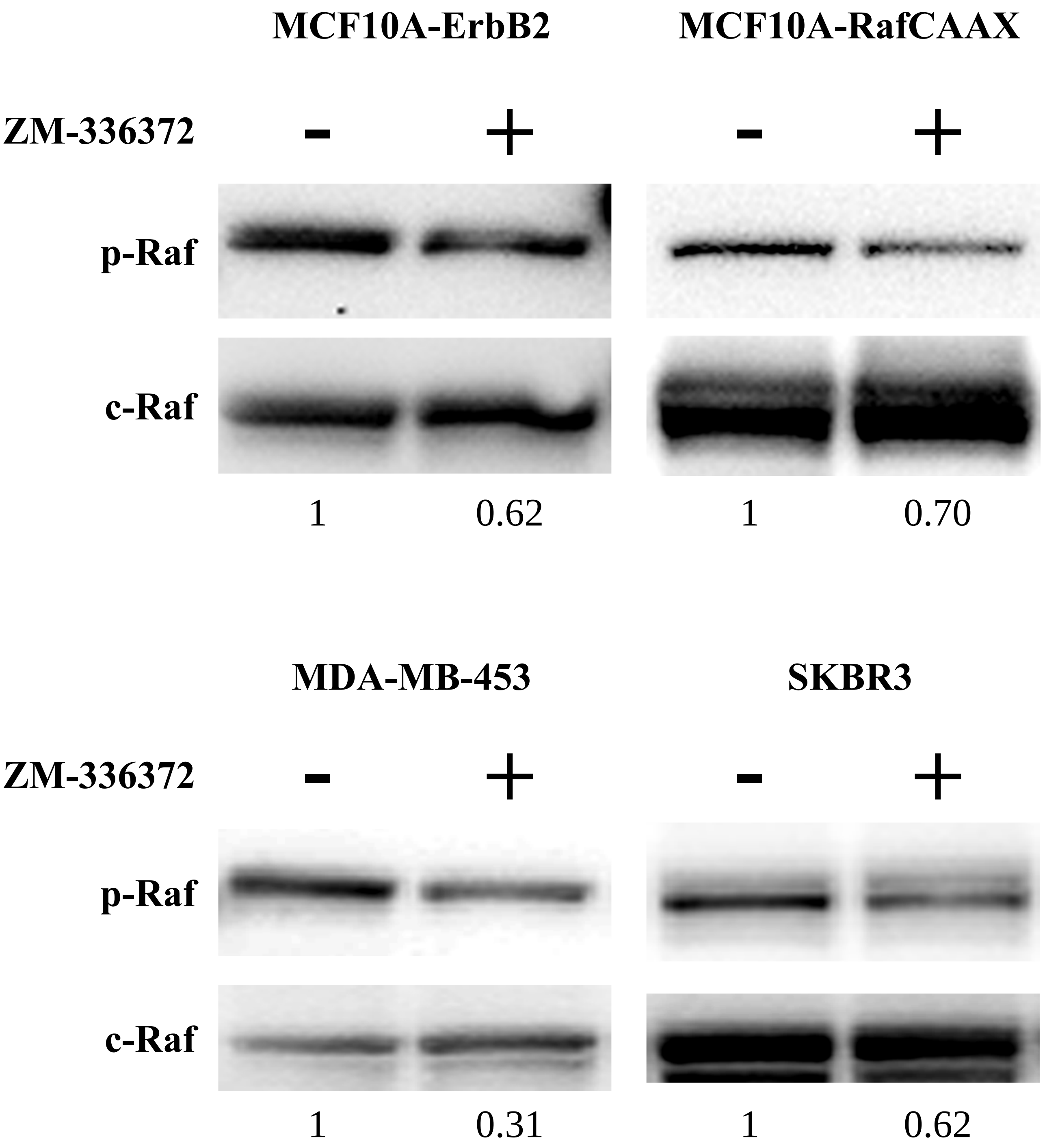

Supplementary Figure S4

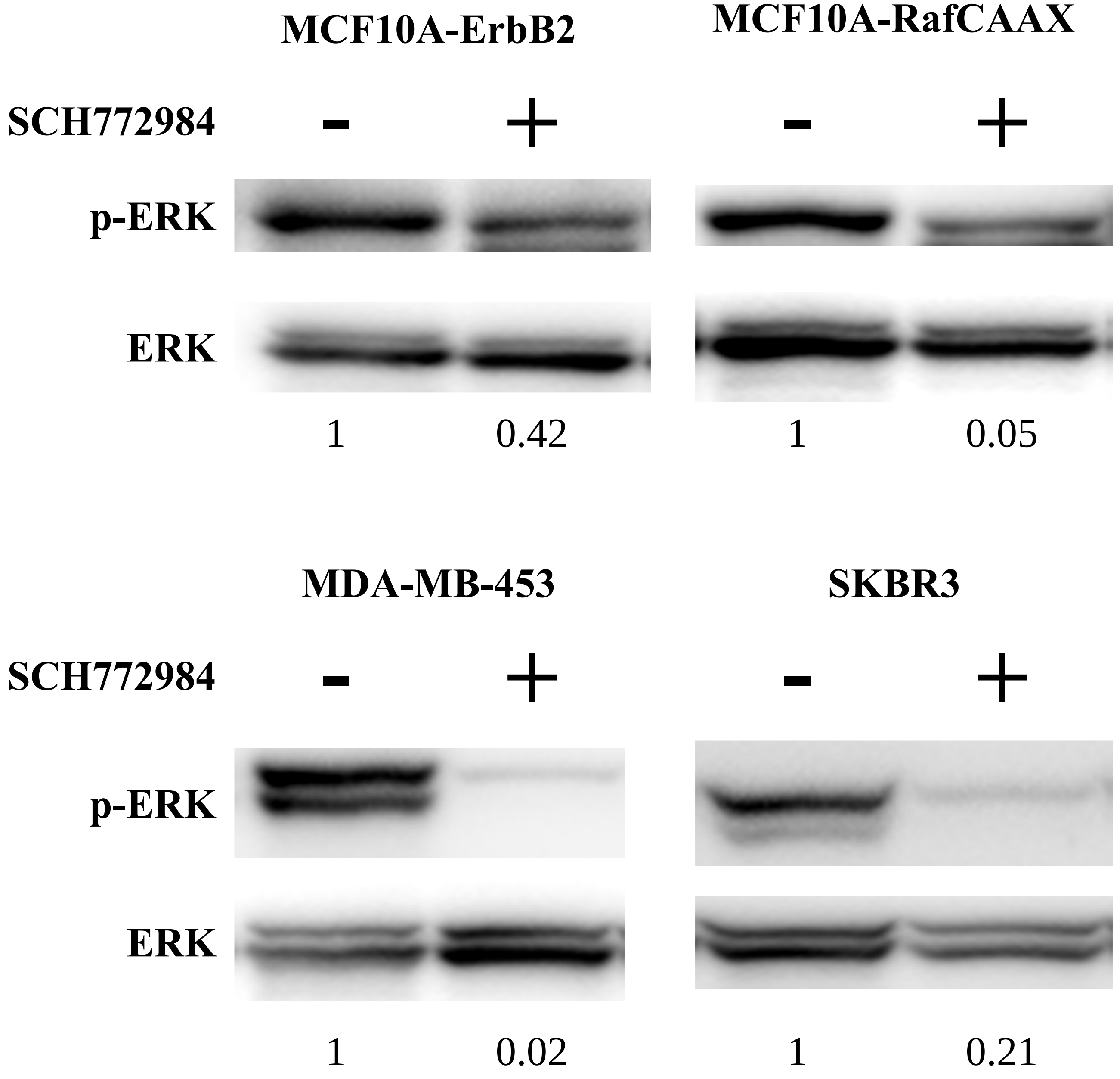

Supplementary Figure S5

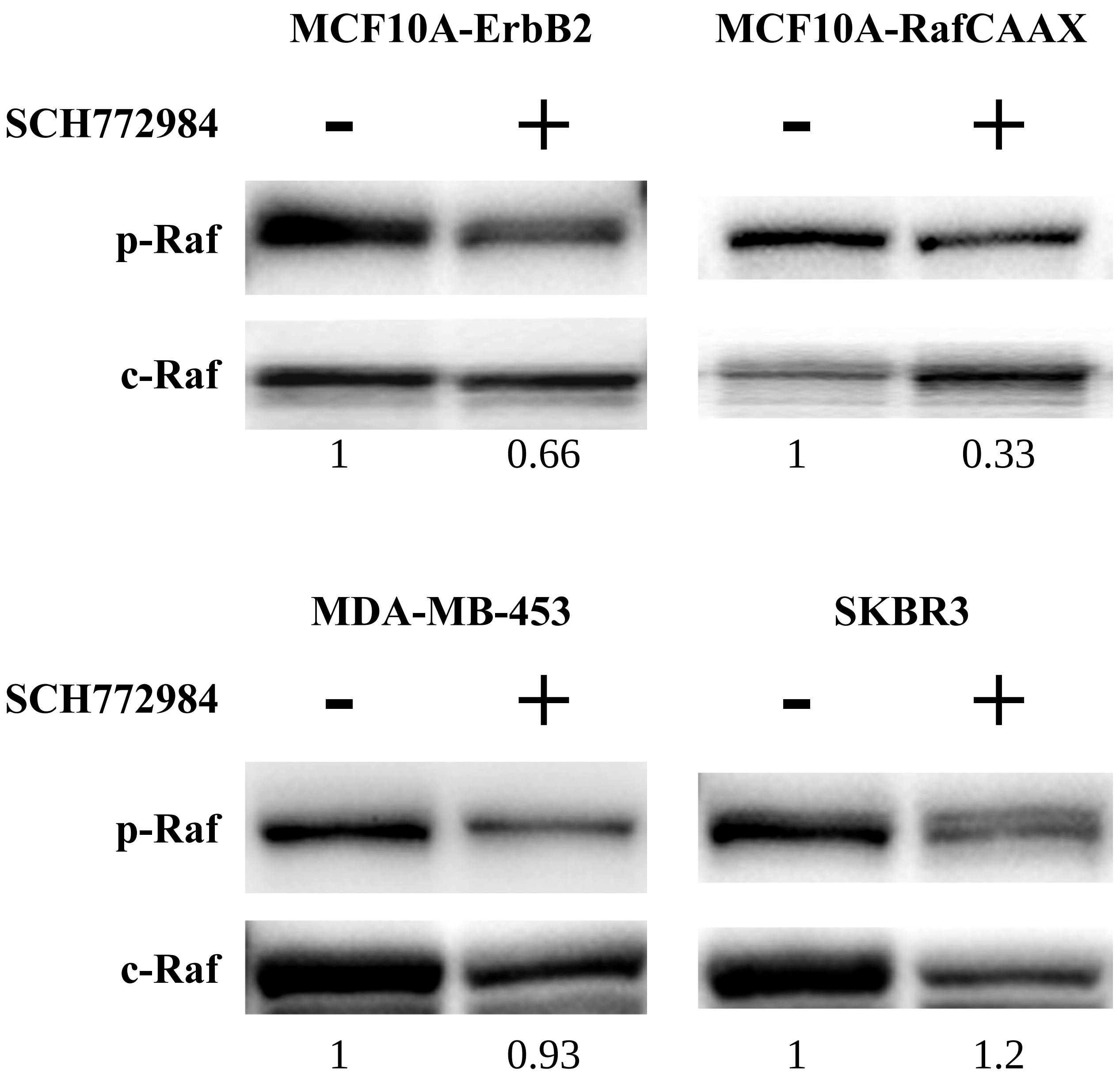

Supplement: Supplementary file 1 — Fig. S1. ErbB2 expression in MCF10A, SKBR3 and MDA‐MB‐453 cells. Fig. S2. Effects of PD98059 or U0126 in MCF10A‐ErbB2, MCF10A‐RafCAAX, MDA‐MB‐453 and SKBR3 cells. Fig. S3. Effects of ZM‐336372 in MCF10A‐ErbB2, MCF10A‐RafCAAX, MDA‐MB‐453 and SKBR3 cells. Fig. S4. Effects of SCH772984 on ERK phosphorylation in MCF10A‐ErbB2, MCF10A‐RafCAAX, MDA‐MB‐453 and SKBR3 cells. Fig. S5. Effects of SCH772984 on c‐Raf phosphorylation in MCF10A‐ErbB2, MCF10A‐RafCAAX, MDA‐MB‐453 and SKBR3 cells. [file FEB4-7-1154-s001.pdf]
